# Supplementary material for: Sequence, structure prediction, and epitope analysis of the polymorphic membrane protein family in Chlamydia trachomatis
Source: PLoS One. 2024 Jun 11;19(6):e0304525. doi: 10.1371/journal.pone.0304525 (PMC11166332; doi:10.1371/journal.pone.0304525)
Supplement: Attachment — Submitted filename: Response to Reviewers.pdf [file pone.0304525.s010.pdf]

Reviewer #1: The manuscript by Cervantes et al provides an excellent description of in-silico-based analysis of the chlamydial Pmp family of proteins. The depth of analysis is considerable and the paper is well written. I have no significant criticisms of the work. The molecular dynamics, epitope prediction, and hinge analysis are particularly unique.

A similar paper has recently been published that describes an Alphafold-based analysis of this family of proteins. This manuscript and that paper look at the same issue from slightly different perspectives and both will be useful to the research community. It is suggested that these authors cite the other manuscript and perhaps briefly describe differences between the documents, with a goal of helping researchers get a wider perspective on predicted Pmp structure. There are data in that manuscript that differ slightly from this one, and certain subjects are addressed by one and not the other one. Both documents will be valuable to people who wish to explore chlamydial Pmp structure.

> We thank the reviewer for pointing out this paper. We had actually shared a copy of the preprint of our manuscript with the authors earlier, but had missed the publication of their paper. We have added a number of references throughout the manuscript.

Minor comments about the writing:

Lines 183 vs. 185, and elsewhere… the designation of I,L,V or ILV needs to be consistent.

> After reviewing dozens of papers on the Pmp repeat motifs, we found that "GGA(I,L,V)" is the most common notation used in text, and we have tried to use it consistently throughout the paper. We did correct one instance on line 74. On line 185, we list the corresponding pattern in PROSITE notation to be used on the ScanProsite search engine, which does look significantly different. We have edited that line to better explain the difference in notation.

Perhaps the MOMP presentation in the Introduction is too drawn out. Lines 32-43 could probably be condensed to one or two sentences.

> We have left out the mention of immunotyping using MOMP, which is not really relevant here.

Lines 80-83: A distinction between the sequence similarity in the beta barrel vs the beta helical domain, across the Pmp proteins, might be useful here.

> We've updated the text to clarify that the majority of Pmps (except PmpB vs PmpC) share less than 30% amino acid identity, and that sequence identity is similar in the beta barrel versus the passenger domain. We tend to think of the beta barrel as well conserved, but that is mainly because there is a reliable Pfam domain for it.

Reviewer #2: Review of MS PONE-D-23-36013 by Cervantes et al.

This MS utilizes protein structure prediction algorithms to visualize, for the first time, the Pmp family from C. trachomatis Serovar E. A special focus has been put onto the tetrapeptide motifs, GGA(I,L,V) and FxxN.

The MS is interesting and adds to our understanding of Pmp protein function. My main critics is that the authors do not try to compare their Pmp structures with published functional data on Pmp proteins and protein fragments. We have published for C. pneumoniae Pmp21, that different protein fragments have different adhesion capacities and that protein fragments with 2 motifs, when point-mutated, lose this capacity (Mölleken et al ). Our study on C. trachomatis Pmps shows that certain motif-rich and motif-poor Pmp protein fragments have a different capacity for adhesion and oligomerization (Favaroni 2021). Finally, we have used large segments of certain C. trachomatis Pmp proteins for a vaccine study (Lanfermann et al; doi.org/10.3390/vaccines9060609). Finally, our biochemical work on a Pmp21 protein fragment has revealed that this protein domain forms oligomers that adopt an amyloid-like structure comparable in size and shape to protofibrils of the amyloid protein Abeta42 (Luczak et al). It would be really interesting to compare the detailed structures presented in this MS with some of the functional data published.

> We have included a section on Pmp21-D, using ESMfold to predict the effect of the FxxN mutations on the structure, AlphaFold-MULTIMER to predict how this may affect oligomerization, and DREAMM to show the effect on a predicted membrane-interacting tyrosine within the first mutated FxxN.
>
> We had hoped to find a simple oligomerization mechanism across all Pmp passenger domain fragments, like the one observed for the H.influenza Hap adhesin (see https://www.ncbi.nlm.nih.gov/pmc/articles/PMC3173798/figure/f5/). AlphaFold-MULTIMER predictions of oligomer structure of the PmpA-PmpI passenger domains, as well as the Pmp21, PmpA, PmpD, PmpG and PmpI fragments described in these papers do not tend to yield a consistent oligomerization pattern. Out of the 34 AlphaFold-MULTIMER dimer, trimer and tetramer runs, only 10 yielded moderate to good confidence structures (pTM = 0.685 – 0.874), including 7 where the two beta-helices were in line with each other (4 C-terminal to C-terminal, 2 N-terminal to N-terminal, and 1 N-terminal to C-terminal), and 3 with the beta-helices next to each other (1 parallel, 2 antiparallel). Interestingly, the PmpA+G, PmpA+I, and especially PmpA+D heterodimers from Favaroni 2021 all show higher model confidence scores than their respective homodimers, in line with the oligomerization results from that paper. Also note that the two "motif-poor" Pmp protein fragments in Favaroni 2021 (A and G) are actually predicted to have an equivalent number of beta-helical coils as the "motif-poor" ones, with an average of around 38 amino acids per coil. The main difference lies in the fact that the "motif-poor" fragments are shorter, and overlap the Middle domain, which has a different sequence specificity (but still largely beta-helical structure). We have included a figure with the Pmp21-D predicted tetramer model, and the two most high-confidence dimer models (the PmpA+D heterodimer, and PmpE homodimer).

The introduction is detailed but needs a few corrections:

Line 47: "….viable vaccine candidates…". The authors missed one recent publication which convincingly shows that a mix of different recombinant C. trachomatis Pmps induces a high degree of cross-serovar protection against urogenital and ocular strains of C. trachomatis: Lanfermann et al., DOI: 10.3390/vaccines9060609.

Reference has been added

Line 56: “Pmps are autotransporters”. That is not true. Pmps are predicted to be autotransporters. So far no experimental prove. Needs to be corrected.

Corrected.

Fig. 6 shows a molecular dynamics simulation of PmpE with the β-barrel domain embedded in a POPC bilayer. I am wondering whether the simulation gives a similar result, when the authors take into account that a typical outer membrane of a G- - negative bacterium is very asymmetrical, with phospholipids in the inner leaflet and LPS in the outer leaflet, plus all of the transmembrane proteins with their β-barrel fold (outer membrane proteins (OMPs)) and the lipoproteins.

The main purpose of doing the molecular dynamics simulations was to explain why many of the structure predictions show the passenger domain at a physiologically unrealistic sharp angle to the beta barrel, where it would intersect the membrane if the beta barrel was embedded in the OM. Clearly the presence of LPS and abundant transmembrane proteins such as MOMP would affect the angle as well, but such simulations would be much more expensive and may not add much additional insight to the topic in this manuscript. We hope to include some of this in a future paper on the Chlamydia Outer Membrane Complex.

Fig. 7. I could not find a figure legend.

We have reinserted the missing figure legend.

Line 359. The authors define proline-rich regions (PRRs) as having at least a P-X0-2-P motive (see Fig. 9 legend). What is the basis of this definition?

Fig. 9. The legend says: “…A few proline-rich regions in side loops of PmpC, PmpG, PmpH and PmpI are labeled.” . To make this point stronger, the authors should label all proline-rich regions - not only a few.

We were not able to find a unique definition of what constitutes the minimum requirement for a proline-rich region. Examining the wide range of different repeating and non-repeating proline-rich regions listed in Williamson 1994, the vast majority seem to contain at least four prolines, separated by no more than four other amino acids. We’ve decided to adopt this as our criterion for Figure 9, which also allows us to label all the corresponding regions on that figure.
