# Supplementary material for: Sequence, structure prediction, and epitope analysis of the polymorphic membrane protein family in Chlamydia trachomatis
Source: PLoS One. 2024 Jun 11;19(6):e0304525. doi: 10.1371/journal.pone.0304525 (PMC11166332; doi:10.1371/journal.pone.0304525)
Supplement: Attachment — Submitted filename: Response to Reviewers2.docx [file pone.0304525.s011.docx]

Reviewer #2:

In the revised MS, the authors dealt satisfactorily with all the points I had raised. In particular, I like the data and the discussion about the oligomerization potential of Pmp proteins.

One important aspect I did not mention in my first review concerns the terminology: The authors use a structure prediction program as a basis for their work. Therefore, all conclusions from these structural predictions are also predictions and not experimentally documented facts. This should be clearly indicated throughout the MS, e.g.

- the title of this MS omits this fact,

- Line 11. Under ´Keywords` no mentioning of the use of a structure prediction program….

Note that structure prediction was explicitly mentioned in the 2^nd^ and 3^rd^ sentences of the abstract. But for completeness, we have included it in the title and keywords, as suggested.

- in the second line of the abstract (lines 15-16) the authors state “Here we show that the 16 characteristic GGA(I,L,V) and FxxN tetrapeptide repeats fit into a larger repeat sequence….” . Up to here, no mentioning of a structure prediction program. I suggest starting the abstract with sentence # three of the current abstract (Analysis of the protein using structure prediction algorithms provided novel insight to the chlamydial Pmp family of proteins.).

The analysis of the tetrapepdtide motifs (section 4.1), showing that they fit into a longer repeat pattern is indeed purely sequence based. It is rather surprising that this was not caught by Grimwood and Stephens back in 1999 when they claimed that the motifs did “not appear to follow any characteristic spacing pattern”, because to our eyes the alternating pattern and semi-regular spacing on Pmp sequence maps was quite striking. We feel that the flow of the manuscript works better by starting with the purely sequence based analysis, then follow it up with how the longer sequence repeat pattern that we identified matches with the rungs of the beta-helix in the predicted structures. We decided to keep the order in the abstract the same as the order in which these analyses are presented in the Results section.

- line 23. The authors state `This structural analysis….` . It is a predicted structure…

We have omitted “structural” here, because the analysis is based both on sequence and predicted structure

Minor points:

- Line 54. The Greek symbol for beta is missing.

We could not find a missing beta symbol on line 54, or anywhere else in this paragraph.

- Figures 1+2. Some of the text in the figures are not readable…..

We recreated Fig 1 by hand in vector graphics with slightly larger font sizes, so the text should be both larger and sharper. Likewise, we recreated the table with HMM occupancy / insertion frequencies and insertion lengths in Fig 2C and 2D. The reader may still need to download the full size image if they really want to read the HMM weights, but at least the numbers are larger and far more crisp in the new version.
