# Supplementary material for: Sequence, structure prediction, and epitope analysis of the polymorphic membrane protein family in Chlamydia trachomatis
Source: PLoS One. 2024 Jun 11;19(6):e0304525. doi: 10.1371/journal.pone.0304525 (PMC11166332; doi:10.1371/journal.pone.0304525)
Supplement: Peer review history

# Peer review: Decision Letter 0

**DOI:** 10.1371/journal.pone.0304525.r001

**Authors:** Agathe Subtil

11 Dec 2023

PONE-D-23-36013Sequence, Structure, and Epitope Analysis of the Polymorphic Membrane Protein Family in Chlamydia trachomatisPLOS ONE

Dear Dr. D'haeseleer,

Thank you for submitting your manuscript to Traffic. Your study has been sent to two referees for evaluation, and their reports are enclosed below. As you can see, while both referees consider the findings interesting, they also raise important points that need to be addressed before they can support publication in PLoS One. The paper by Debrine et al (doi: 10.1128/spectrum.03242-23), that was published before you submitted your MS, needs to be acknowledged. It will be useful for readers to state clearly in the discussion whether your conclusions differ or not, considering that the same prediction tools have been used. Also, as stated by reviewer 2, it is expected in the discussion (or in the results section, if helps keeping the flow) that you validate (or not) some of your structural predictions based on published functional work.

Please submit your revised manuscript by Jan 25 2024 11:59PM. If you will need more time than this to complete your revisions, please reply to this message or contact the journal office at plosone@plos.org. When you're ready to submit your revision, log on to https://www.editorialmanager.com/pone/ and select the 'Submissions Needing Revision' folder to locate your manuscript file.

Please include the following items when submitting your revised manuscript:

- A rebuttal letter that responds to each point raised by the academic editor and reviewer(s). You should upload this letter as a separate file labeled 'Response to Reviewers'.
- A marked-up copy of your manuscript that highlights changes made to the original version. You should upload this as a separate file labeled 'Revised Manuscript with Track Changes'.
- An unmarked version of your revised paper without tracked changes. You should upload this as a separate file labeled 'Manuscript'.

If you would like to make changes to your financial disclosure, please include your updated statement in your cover letter. Guidelines for resubmitting your figure files are available below the reviewer comments at the end of this letter.

If applicable, we recommend that you deposit your laboratory protocols in protocols.io to enhance the reproducibility of your results. Protocols.io assigns your protocol its own identifier (DOI) so that it can be cited independently in the future. For instructions see: https://journals.plos.org/plosone/s/submission-guidelines#loc-laboratory-protocols. Additionally, PLOS ONE offers an option for publishing peer-reviewed Lab Protocol articles, which describe protocols hosted on protocols.io. Read more information on sharing protocols at https://plos.org/protocols?utm_medium=editorial-email&utm_source=authorletters&utm_campaign=protocols.

We look forward to receiving your revised manuscript.

Kind regards,

Agathe Subtil

Academic Editor

PLOS ONE

Journal Requirements:

When submitting your revision, we need you to address these additional requirements.

1. Please ensure that your manuscript meets PLOS ONE's style requirements, including those for file naming. The PLOS ONE style templates can be found at 

https://journals.plos.org/plosone/s/file?id=wjVg/PLOSOne_formatting_sample_main_body.pdf and 

https://journals.plos.org/plosone/s/file?id=ba62/PLOSOne_formatting_sample_title_authors_affiliations.pdf

2. Please expand the acronym  NIH/NIAID” (as indicated in your financial disclosure) so that it states the name of your funders in full.

This information should be included in your cover letter; we will change the online submission form on your behalf.

[Note: HTML markup is below. Please do not edit.]

Reviewers' comments:

Reviewer's Responses to Questions

**Comments to the Author**

1. Is the manuscript technically sound, and do the data support the conclusions?

The manuscript must describe a technically sound piece of scientific research with data that supports the conclusions. Experiments must have been conducted rigorously, with appropriate controls, replication, and sample sizes. The conclusions must be drawn appropriately based on the data presented. 

Reviewer #1: Yes

Reviewer #2: Yes

**********

2. Has the statistical analysis been performed appropriately and rigorously? 

Reviewer #1: Yes

Reviewer #2: I Don't Know

**********

3. Have the authors made all data underlying the findings in their manuscript fully available?

The PLOS Data policy requires authors to make all data underlying the findings described in their manuscript fully available without restriction, with rare exception (please refer to the Data Availability Statement in the manuscript PDF file). The data should be provided as part of the manuscript or its supporting information, or deposited to a public repository. For example, in addition to summary statistics, the data points behind means, medians and variance measures should be available. If there are restrictions on publicly sharing data—e.g. participant privacy or use of data from a third party—those must be specified.

Reviewer #1: Yes

Reviewer #2: Yes

**********

4. Is the manuscript presented in an intelligible fashion and written in standard English?

PLOS ONE does not copyedit accepted manuscripts, so the language in submitted articles must be clear, correct, and unambiguous. Any typographical or grammatical errors should be corrected at revision, so please note any specific errors here.

Reviewer #1: Yes

Reviewer #2: Yes

**********

5. Review Comments to the Author

Please use the space provided to explain your answers to the questions above. You may also include additional comments for the author, including concerns about dual publication, research ethics, or publication ethics. (Please upload your review as an attachment if it exceeds 20,000 characters)

Reviewer #1: The manuscript by Cervantes et al provides an excellent description of in-silico-based analysis of the chlamydial Pmp family of proteins. The depth of analysis is considerable and the paper is well written. I have no significant criticisms of the work. The molecular dynamics, epitope prediction, and hinge analysis are particularly unique.

A similar paper has recently been published that describes an Alphafold-based analysis of this family of proteins. This manuscript and that paper look at the same issue from slightly different perspectives and both will be useful to the research community. It is suggested that these authors cite the other manuscript and perhaps briefly describe differences between the documents, with a goal of helping researchers get a wider perspective on predicted Pmp structure. There are data in that manuscript that differ slightly from this one, and certain subjects are addressed by one and not the other one. Both documents will be valuable to people who wish to explore chlamydial Pmp structure.

Minor comments about the writing:

Lines 183 vs. 185, and elsewhere… the designation of I,L,V or ILV needs to be consistent.

Perhaps the MOMP presentation in the Introduction is too drawn out. Lines 32-43 could probably be condensed to one or two sentences.

Lines 80-83: A distinction between the sequence similarity in the beta barrel vs the beta helical domain, across the Pmp proteins, might be useful here.

Reviewer #2: Review of MS PONE-D-23-36013 by Cervantes et al.

This MS utilizes protein structure prediction algorithms to visualize, for the first time, the Pmp family from C. trachomatis Serovar E. A special focus has been put onto the tetrapeptide motifs, GGA(I,L,V) and FxxN.

The MS is interesting and adds to our understanding of Pmp protein function. My main critics is that the authors do not try to compare their Pmp structures with published functional data on Pmp proteins and protein fragments. We have published for C. pneumoniae Pmp21, that different protein fragments have different adhesion capacities and that protein fragments with 2 motifs, when point-mutated, lose this capacity (Mölleken et al ). Our study on C. trachomatis Pmps shows that certain motif-rich and motif-poor Pmp protein fragments have a different capacity for adhesion and oligomerization (Favaroni 2021). Finally, we have used large segments of certain C. trachomatis Pmp proteins for a vaccine study (Lanfermann et al; doi.org/10.3390/vaccines9060609). Finally, our biochemical work on a Pmp21 protein fragment has revealed that this protein domain forms oligomers that adopt an amyloid-like structure comparable in size and shape to protofibrils of the amyloid protein Abeta42 (Luczak et al). It would be really interesting to compare the detailed structures presented in this MS with some of the functional data published.

The introduction is detailed but needs a few corrections:

Line 47: “….viable vaccine candidates…”. The authors missed one recent publication which convincingly shows that a mix of different recombinant C. trachomatis Pmps induces a high degree of cross-serovar protection against urogenital and ocular strains of C. trachomatis: Lanfermann et al., DOI: 10.3390/vaccines9060609.

Line 56: “Pmps are autotransporters”. That is not true. Pmps are predicted to be autotransporters. So far no experimental prove. Needs to be corrected.

Fig. 6 shows a molecular dynamics simulation of PmpE with the β-barrel domain embedded in a POPC bilayer. I am wondering whether the simulation gives a similar result, when the authors take into account that a typical outer membrane of a G- - negative bacterium is very asymmetrical, with phospholipids in the inner leaflet and LPS in the outer leaflet, plus all of the transmembrane proteins with their β-barrel fold (outer membrane proteins (OMPs)) and the lipoproteins.

Fig. 7. I could not find a figure legend.

Line 359. The authors define proline-rich regions (PRRs) as having at least a P-X0-2-P motive (see Fig. 9 legend). What is the basis of this definition?

Fig. 9. The legend says: “…A few proline-rich regions in side loops of PmpC, PmpG, PmpH and PmpI are labeled.” . To make this point stronger, the authors should label all proline-rich regions - not only a few.

Johannes Hegemann

**********

6. PLOS authors have the option to publish the peer review history of their article (what does this mean?). If published, this will include your full peer review and any attached files.

If you choose “no”, your identity will remain anonymous but your review may still be made public.

**Do you want your identity to be public for this peer review?** For information about this choice, including consent withdrawal, please see our Privacy Policy.

Reviewer #1: No

Reviewer #2: **Yes: **Johannes H. Hegemann

**********

[NOTE: If reviewer comments were submitted as an attachment file, they will be attached to this email and accessible via the submission site. Please log into your account, locate the manuscript record, and check for the action link "View Attachments". If this link does not appear, there are no attachment files.]

While revising your submission, please upload your figure files to the Preflight Analysis and Conversion Engine (PACE) digital diagnostic tool, https://pacev2.apexcovantage.com/. PACE helps ensure that figures meet PLOS requirements. To use PACE, you must first register as a user. Registration is free. Then, login and navigate to the UPLOAD tab, where you will find detailed instructions on how to use the tool. If you encounter any issues or have any questions when using PACE, please email PLOS at figures@plos.org. Please note that Supporting Information files do not need this step.

# Author comment: Author response to Decision Letter 0

**DOI:** 10.1371/journal.pone.0304525.r002

6 Mar 2024

Reviewer #1: The manuscript by Cervantes et al provides an excellent description of in-silico-based analysis of the chlamydial Pmp family of proteins. The depth of analysis is considerable and the paper is well written. I have no significant criticisms of the work. The molecular dynamics, epitope prediction, and hinge analysis are particularly unique.

A similar paper has recently been published that describes an Alphafold-based analysis of this family of proteins. This manuscript and that paper look at the same issue from slightly different perspectives and both will be useful to the research community. It is suggested that these authors cite the other manuscript and perhaps briefly describe differences between the documents, with a goal of helping researchers get a wider perspective on predicted Pmp structure. There are data in that manuscript that differ slightly from this one, and certain subjects are addressed by one and not the other one. Both documents will be valuable to people who wish to explore chlamydial Pmp structure.

We thank the reviewer for pointing out this paper. We had actually shared a copy of the preprint of our manuscript with the authors earlier, but had missed the publication of their paper. We have added a number of references throughout the manuscript. 

Minor comments about the writing:

Lines 183 vs. 185, and elsewhere… the designation of I,L,V or ILV needs to be consistent.

After reviewing dozens of papers on the Pmp repeat motifs, we found that “GGA(I,L,V)” is the most common notation used in text, and we have tried to use it consistently throughout the paper. We did correct one instance on line 74. On line 185, we list the corresponding pattern in PROSITE notation to be used on the ScanProsite search engine, which does look significantly different. We have edited that line to better explain the difference in notation.

Perhaps the MOMP presentation in the Introduction is too drawn out. Lines 32-43 could probably be condensed to one or two sentences.

We have left out the mention of immunotyping using MOMP, which is not really relevant here. 

Lines 80-83: A distinction between the sequence similarity in the beta barrel vs the beta helical domain, across the Pmp proteins, might be useful here.

We’ve updated the text to clarify that the majority of Pmps (except PmpB vs PmpC) share less than 30% amino acid identity, and that sequence identity is similar in the beta barrel versus the passenger domain. We tend to think of the beta barrel as well conserved, but that is mainly because there is a reliable Pfam domain for it. 

Reviewer #2: Review of MS PONE-D-23-36013 by Cervantes et al.

This MS utilizes protein structure prediction algorithms to visualize, for the first time, the Pmp family from C. trachomatis Serovar E. A special focus has been put onto the tetrapeptide motifs, GGA(I,L,V) and FxxN.

The MS is interesting and adds to our understanding of Pmp protein function. My main critics is that the authors do not try to compare their Pmp structures with published functional data on Pmp proteins and protein fragments. We have published for C. pneumoniae Pmp21, that different protein fragments have different adhesion capacities and that protein fragments with 2 motifs, when point-mutated, lose this capacity (Mölleken et al ). Our study on C. trachomatis Pmps shows that certain motif-rich and motif-poor Pmp protein fragments have a different capacity for adhesion and oligomerization (Favaroni 2021). Finally, we have used large segments of certain C. trachomatis Pmp proteins for a vaccine study (Lanfermann et al; doi.org/10.3390/vaccines9060609). Finally, our biochemical work on a Pmp21 protein fragment has revealed that this protein domain forms oligomers that adopt an amyloid-like structure comparable in size and shape to protofibrils of the amyloid protein Abeta42 (Luczak et al). It would be really interesting to compare the detailed structures presented in this MS with some of the functional data published.

We have included a section on Pmp21-D, using ESMfold to predict the effect of the FxxN mutations on the structure, AlphaFold-MULTIMER to predict how this may affect oligomerization, and DREAMM to show the effect on a predicted membrane-interacting tyrosine within the first mutated FxxN. 

We had hoped to find a simple oligomerization mechanism across all Pmp passenger domain fragments, like the one observed for the H.influenza Hap adhesin (see https://www.ncbi.nlm.nih.gov/pmc/articles/PMC3173798/figure/f5/). AlphaFold-MULTIMER predictions of oligomer structure of the PmpA-PmpI passenger domains, as well as the Pmp21, PmpA, PmpD, PmpG and PmpI fragments described in these papers do not tend to yield a consistent oligomerization pattern. Out of the 34 AlphaFold-MULTIMER dimer, trimer and tetramer runs, only 10 yielded moderate to good confidence structures (pTM = 0.685 – 0.874), including 7 where the two beta-helices were in line with each other (4 C-terminal to C-terminal, 2 N-terminal to N-terminal, and 1 N-terminal to C-terminal), and 3 with the beta-helices next to each other (1 parallel, 2 antiparallel). Interestingly, the PmpA+G, PmpA+I, and especially PmpA+D heterodimers from Favaroni 2021 all show higher model confidence scores than their respective homodimers, in line with the oligomerization results from that paper. Also note that the two “motif-poor” Pmp protein fragments in Favaroni 2021 (A and G) are actually predicted to have an equivalent number of beta-helical coils as the “motif-poor” ones, with an average of around 38 amino acids per coil. The main difference lies in the fact that the “motif-poor” fragments are shorter, and overlap the Middle domain, which has a different sequence specificity (but still largely beta-helical structure). We have included a figure with the Pmp21-D predicted tetramer model, and the two most high-confidence dimer models (the PmpA+D heterodimer, and PmpE homodimer).

The introduction is detailed but needs a few corrections:

Line 47: “….viable vaccine candidates…”. The authors missed one recent publication which convincingly shows that a mix of different recombinant C. trachomatis Pmps induces a high degree of cross-serovar protection against urogenital and ocular strains of C. trachomatis: Lanfermann et al., DOI: 10.3390/vaccines9060609.

 Reference has been added 

Line 56: “Pmps are autotransporters”. That is not true. Pmps are predicted to be autotransporters. So far no experimental prove. Needs to be corrected.

Corrected.

Fig. 6 shows a molecular dynamics simulation of PmpE with the β-barrel domain embedded in a POPC bilayer. I am wondering whether the simulation gives a similar result, when the authors take into account that a typical outer membrane of a G- - negative bacterium is very asymmetrical, with phospholipids in the inner leaflet and LPS in the outer leaflet, plus all of the transmembrane proteins with their β-barrel fold (outer membrane proteins (OMPs)) and the lipoproteins.

The main purpose of doing the molecular dynamics simulations was to explain why many of the structure predictions show the passenger domain at a physiologically unrealistic sharp angle to the beta barrel, where it would intersect the membrane if the beta barrel was embedded in the OM. Clearly the presence of LPS and abundant transmembrane proteins such as MOMP would affect the angle as well, but such simulations would be much more expensive and may not add much additional insight to the topic in this manuscript. We hope to include some of this in a future paper on the Chlamydia Outer Membrane Complex. 

Fig. 7. I could not find a figure legend.

We have reinserted the missing figure legend.

Line 359. The authors define proline-rich regions (PRRs) as having at least a P-X0-2-P motive (see Fig. 9 legend). What is the basis of this definition?

Fig. 9. The legend says: “…A few proline-rich regions in side loops of PmpC, PmpG, PmpH and PmpI are labeled.” . To make this point stronger, the authors should label all proline-rich regions - not only a few.

We were not able to find a unique definition of what constitutes the minimum requirement for a proline-rich region. Examining the wide range of different repeating and non-repeating proline-rich regions listed in Williamson 1994, the vast majority seem to contain at least four prolines, separated by no more than four other amino acids. We’ve decided to adopt this as our criterion for Figure 9, which also allows us to label all the corresponding regions on that figure.

Attachment Submitted filename: Response to Reviewers.pdf

# Peer review: Decision Letter 1

**DOI:** 10.1371/journal.pone.0304525.r003

**Authors:** Agathe Subtil

16 Apr 2024

PONE-D-23-36013R1Sequence, Structure, and Epitope Analysis of the Polymorphic Membrane Protein Family in Chlamydia trachomatisPLOS ONE

Dear Dr. D'haeseleer,

Thank you for submitting your revised manuscript to PLOS ONE. You have satisfactorily responded to all the reviewers's comments. However, reviewer 2 made a finale point that I would appreciate you to address by sending a revised version (very minor revision).  

If you would like to make changes to your financial disclosure, please include your updated statement in your cover letter. Guidelines for resubmitting your figure files are available below the reviewer comments at the end of this letter.

Please submit your revision by May 31 2024 11:59PM.

If applicable, we recommend that you deposit your laboratory protocols in protocols.io to enhance the reproducibility of your results. Protocols.io assigns your protocol its own identifier (DOI) so that it can be cited independently in the future. For instructions see: https://journals.plos.org/plosone/s/submission-guidelines#loc-laboratory-protocols. Additionally, PLOS ONE offers an option for publishing peer-reviewed Lab Protocol articles, which describe protocols hosted on protocols.io. Read more information on sharing protocols at https://plos.org/protocols?utm_medium=editorial-email&utm_source=authorletters&utm_campaign=protocols.

We look forward to receiving your revised manuscript.

Kind regards,

Agathe Subtil

Academic Editor

PLOS ONE

Journal Requirements:

Please review your reference list to ensure that it is complete and correct. If you have cited papers that have been retracted, please include the rationale for doing so in the manuscript text, or remove these references and replace them with relevant current references. Any changes to the reference list should be mentioned in the rebuttal letter that accompanies your revised manuscript. If you need to cite a retracted article, indicate the article’s retracted status in the References list and also include a citation and full reference for the retraction notice.

Additional Editor Comments:

Congratulation for this paper. PLease take into account the comments from reviewer 2 for minor revision of your manuscript.

[Note: HTML markup is below. Please do not edit.]

Reviewers' comments:

Reviewer's Responses to Questions

**Comments to the Author**

1. If the authors have adequately addressed your comments raised in a previous round of review and you feel that this manuscript is now acceptable for publication, you may indicate that here to bypass the “Comments to the Author” section, enter your conflict of interest statement in the “Confidential to Editor” section, and submit your "Accept" recommendation.

Reviewer #2: All comments have been addressed

**********

2. Is the manuscript technically sound, and do the data support the conclusions?

The manuscript must describe a technically sound piece of scientific research with data that supports the conclusions. Experiments must have been conducted rigorously, with appropriate controls, replication, and sample sizes. The conclusions must be drawn appropriately based on the data presented. 

Reviewer #2: Yes

**********

3. Has the statistical analysis been performed appropriately and rigorously? 

Reviewer #2: I Don't Know

**********

4. Have the authors made all data underlying the findings in their manuscript fully available?

The PLOS Data policy requires authors to make all data underlying the findings described in their manuscript fully available without restriction, with rare exception (please refer to the Data Availability Statement in the manuscript PDF file). The data should be provided as part of the manuscript or its supporting information, or deposited to a public repository. For example, in addition to summary statistics, the data points behind means, medians and variance measures should be available. If there are restrictions on publicly sharing data—e.g. participant privacy or use of data from a third party—those must be specified.

Reviewer #2: Yes

**********

5. Is the manuscript presented in an intelligible fashion and written in standard English?

PLOS ONE does not copyedit accepted manuscripts, so the language in submitted articles must be clear, correct, and unambiguous. Any typographical or grammatical errors should be corrected at revision, so please note any specific errors here.

Reviewer #2: Yes

**********

6. Review Comments to the Author

Please use the space provided to explain your answers to the questions above. You may also include additional comments for the author, including concerns about dual publication, research ethics, or publication ethics. (Please upload your review as an attachment if it exceeds 20,000 characters)

Reviewer #2: Review of MS PONE-D-23-36013_R1 by Cervantes et al.

In the revised MS, the authors dealt satisfactorily with all the points I had raised. In particular, I like the data and the discussion about the oligomerization potential of Pmp proteins.

One important aspect I did not mention in my first review concerns the terminology: The authors use a structure prediction program as a basis for their work. Therefore, all conclusions from these structural predictions are also predictions and not experimentally documented facts. This should be clearly indicated throughout the MS, e.g.

- the title of this MS omits this fact,

- Line 11. Under ´Keywords` no mentioning of the use of a structure prediction program….

- in the second line of the abstract (lines 15-16) the authors state “Here we show that the 16 characteristic GGA(I,L,V) and FxxN tetrapeptide repeats fit into a larger repeat sequence….” . Up to here, no mentioning of a structure prediction program. I suggest starting the abstract with sentence # three of the current abstract (Analysis of the protein using structure prediction algorithms provided novel insight to the chlamydial Pmp family of proteins.).

- line 23. The authors state `This structural analysis….` . It is a predicted structure…

Minor points:

- Line 54. The Greek symbol for beta is missing.

- Figures 1+2. Some of the text in the figures are not readable…..

Johannes Hegemann

**********

7. PLOS authors have the option to publish the peer review history of their article (what does this mean?). If published, this will include your full peer review and any attached files.

If you choose “no”, your identity will remain anonymous but your review may still be made public.

**Do you want your identity to be public for this peer review?** For information about this choice, including consent withdrawal, please see our Privacy Policy.

Reviewer #2: **Yes: **Johannes Hegemann

**********

[NOTE: If reviewer comments were submitted as an attachment file, they will be attached to this email and accessible via the submission site. Please log into your account, locate the manuscript record, and check for the action link "View Attachments". If this link does not appear, there are no attachment files.]

While revising your submission, please upload your figure files to the Preflight Analysis and Conversion Engine (PACE) digital diagnostic tool, https://pacev2.apexcovantage.com/. PACE helps ensure that figures meet PLOS requirements. To use PACE, you must first register as a user. Registration is free. Then, login and navigate to the UPLOAD tab, where you will find detailed instructions on how to use the tool. If you encounter any issues or have any questions when using PACE, please email PLOS at figures@plos.org. Please note that Supporting Information files do not need this step.

# Author comment: Author response to Decision Letter 1

**DOI:** 10.1371/journal.pone.0304525.r004

11 May 2024

Reviewer #2:

In the revised MS, the authors dealt satisfactorily with all the points I had raised. In particular, I like the data and the discussion about the oligomerization potential of Pmp proteins.

One important aspect I did not mention in my first review concerns the terminology: The authors use a structure prediction program as a basis for their work. Therefore, all conclusions from these structural predictions are also predictions and not experimentally documented facts. This should be clearly indicated throughout the MS, e.g.

- the title of this MS omits this fact,

- Line 11. Under ´Keywords` no mentioning of the use of a structure prediction program….

Note that structure prediction was explicitly mentioned in the 2nd and 3rd sentences of the abstract. But for completeness, we have included it in the title and keywords, as suggested.

- in the second line of the abstract (lines 15-16) the authors state “Here we show that the 16 characteristic GGA(I,L,V) and FxxN tetrapeptide repeats fit into a larger repeat sequence….” . Up to here, no mentioning of a structure prediction program. I suggest starting the abstract with sentence # three of the current abstract (Analysis of the protein using structure prediction algorithms provided novel insight to the chlamydial Pmp family of proteins.).

The analysis of the tetrapepdtide motifs (section 4.1), showing that they fit into a longer repeat pattern is indeed purely sequence based. It is rather surprising that this was not caught by Grimwood and Stephens back in 1999 when they claimed that the motifs did “not appear to follow any characteristic spacing pattern”, because to our eyes the alternating pattern and semi-regular spacing on Pmp sequence maps was quite striking. We feel that the flow of the manuscript works better by starting with the purely sequence based analysis, then follow it up with how the longer sequence repeat pattern that we identified matches with the rungs of the beta-helix in the predicted structures. We decided to keep the order in the abstract the same as the order in which these analyses are presented in the Results section.

- line 23. The authors state `This structural analysis….` . It is a predicted structure…

We have omitted “structural” here, because the analysis is based both on sequence and predicted structure

Minor points:

- Line 54. The Greek symbol for beta is missing.

We could not find a missing beta symbol on line 54, or anywhere else in this paragraph.

- Figures 1+2. Some of the text in the figures are not readable…..

We recreated Fig 1 by hand in vector graphics with slightly larger font sizes, so the text should be both larger and sharper. Likewise, we recreated the table with HMM occupancy / insertion frequencies and insertion lengths in the Fig 2C and 2D sequence logos. The reader may still need to download the full size image if they really want to read the HMM weights, but at least the numbers are larger and far more crisp in the new version.

Attachment Submitted filename: Response to Reviewers2.docx

# Editor report: Decision Letter 2

**DOI:** 10.1371/journal.pone.0304525.r005

**Authors:** Agathe Subtil

14 May 2024

Sequence, Structure Prediction, and Epitope Analysis of the Polymorphic Membrane Protein Family in Chlamydia trachomatis

PONE-D-23-36013R2

Dear Dr. D'haeseleer,

We’re pleased to inform you that your manuscript has been judged scientifically suitable for publication and will be formally accepted for publication once it meets all outstanding technical requirements.

Within one week, you’ll receive an e-mail detailing the required amendments. When these have been addressed, you’ll receive a formal acceptance letter and your manuscript will be scheduled for publication.

An invoice will be generated when your article is formally accepted. Please note, if your institution has a publishing partnership with PLOS and your article meets the relevant criteria, all or part of your publication costs will be covered. Please make sure your user information is up-to-date by logging into Editorial Manager at Editorial Manager® and clicking the ‘Update My Information' link at the top of the page. If you have any questions relating to publication charges, please contact our Author Billing department directly at authorbilling@plos.org.

If your institution or institutions have a press office, please notify them about your upcoming paper to help maximize its impact. If they’ll be preparing press materials, please inform our press team as soon as possible -- no later than 48 hours after receiving the formal acceptance. Your manuscript will remain under strict press embargo until 2 pm Eastern Time on the date of publication. For more information, please contact onepress@plos.org.

Kind regards,

Agathe Subtil

Academic Editor

PLOS ONE

Additional Editor Comments (optional):

Reviewers' comments:

# Editor report: Acceptance letter

**DOI:** 10.1371/journal.pone.0304525.r006

**Authors:** Agathe Subtil

31 May 2024

PONE-D-23-36013R2 

PLOS ONE

Dear Dr. D'haeseleer, 

I'm pleased to inform you that your manuscript has been deemed suitable for publication in PLOS ONE. Congratulations! Your manuscript is now being handed over to our production team.

At this stage, our production department will prepare your paper for publication. This includes ensuring the following:

* All references, tables, and figures are properly cited

* All relevant supporting information is included in the manuscript submission,

* There are no issues that prevent the paper from being properly typeset

If revisions are needed, the production department will contact you directly to resolve them. If no revisions are needed, you will receive an email when the publication date has been set. At this time, we do not offer pre-publication proofs to authors during production of the accepted work. Please keep in mind that we are working through a large volume of accepted articles, so please give us a few weeks to review your paper and let you know the next and final steps. 

Lastly, if your institution or institutions have a press office, please let them know about your upcoming paper now to help maximize its impact. If they'll be preparing press materials, please inform our press team within the next 48 hours. Your manuscript will remain under strict press embargo until 2 pm Eastern Time on the date of publication. For more information, please contact onepress@plos.org.

If we can help with anything else, please email us at customercare@plos.org.

Thank you for submitting your work to PLOS ONE and supporting open access. 

Kind regards, 

PLOS ONE Editorial Office Staff

on behalf of

Dr. Agathe Subtil 

Academic Editor

PLOS ONE
